# Arterial Stiffness, Lifestyle Intervention and a Low-Calorie Diet in Morbidly Obese Patients—A Nonrandomized Clinical Trial

**DOI:** 10.1002/oby.20099

**Published:** 2012-10-18

**Authors:** N Nordstrand, E Gjevestad, JK Hertel, LK Johnson, E Saltvedt, J Røislien, J Hjelmesæth

**Affiliations:** 1 Morbid Obesity Center, Vestfold Hospital TrustTønsberg, Norway; 2 Department of Medicine, Vestfold Hospital TrustTønsberg, Norway; 3 Department of Biostatistics, Institute of Basic Medical Sciences, University of OsloNorway; 4 Department of Heart Disease, Haukeland University HospitalBergen, Norway

## Abstract

**Objective:**

Arterial stiffness is an independent predictor of cardiovascular morbidity and mortality. This study aimed to compare the 7-week effect of a low-calorie diet (LCD) and an intensive lifestyle intervention program (ILI) on arterial stiffness in morbidly obese individuals.

**Design and Methods:**

Nonrandomized clinical trial. The LCD provided 900 kcal/day, and participants in the LCD group were instructed to maintain their habitual physical activity level. The ILI included two 90-min supervised training sessions 3 days a week at moderate to high intensity (4-8 METs) and a caloric restriction of 1000 kcal/day.

**Results:**

A total of 179 individuals completed the study, 88 (56 women) in the ILI group and 91 (57 women) in the LCD group. High-fidelity applanation tonometry (Millar®, Sphygmocor®) was used to measure carotid-femoral pulse wave velocity (PWV). After adjustment for relevant confounders, the ILI group had a significantly greater reduction in PWV than the LCD group; −0.4 (−0.6, −0.1) m/s, *P* = 0.004. When compared to the LCD group, the ILI group showed a larger reduction in systolic and diastolic blood pressure −5 (−9, −1) and −5 (−7, −2) mmHg, *P* = 0.038 and *P* ≤ 0.001 respectively, whereas no difference was observed regarding pulse pressure, *P* = 0.661. No significant differences between groups were found regarding the loss of fat mass, *P* = 0.259, but the loss of muscle mass was larger in the LCD group, 0.8 (0.5, 1.1) kg, *P* ≤ 0.001.

**Conclusion:**

Despite the limitations of a nonrandomized design, our findings indicate that for morbidly obese individuals a moderate caloric restriction combined with aerobic physical exercise is associated with a greater decline in PWV than a LCD alone.

## Background

Obesity is a world-wide health concern (1) and imparts a degree of cardiovascular risk similar to that associated with hypertension, hyperlipidemia, and smoking (2). New insights from the National Health and Nutrition Examination Survey indicate that the prevalence of obesity has stabilized during the last decade, but there is evidence that within the obese population, the prevalence of morbid obesity is increasing at a high rate (3, 4).

The cornerstone of obesity management is weight loss. Any intervention with the intention to cause weight loss must tip the balance between energy intake and expenditure to be successful. Official recommendations state that a reduction in energy intake of ∼500 kcal below energy expenditure per day will result in a weight loss of 0.5 kg per week. There is, however, evidence that the simplicity of the shift in energy balance could be misleading and might not be adaptable to a real-life situation. The weight response to caloric restriction is slow, and to reach steady-state weight, an individual must comply with the dietary intervention for a long time. Purely dietary interventions will be more time consuming and demanding in morbidly obese individuals than in less obese individuals (5). It is therefore important to investigate whether or not there are supplementary efforts (other than a strict diet) that can attenuate the cardiovascular risk associated with morbid obesity. Several lines of evidence indicate that aerobic physical activity might decrease cardiovascular risk in obese individuals (6, 7). Whether or not this is true for morbidly obese individuals is unknown.

Arterial stiffness assessed by aortic pulse wave velocity (PWV) is an independent predictor of cardiovascular morbidity and mortality (8), and PWV is increasingly recognized as a valid surrogate endpoint of cardiovascular disease (9, 10). In healthy young adults, arteries stiffen when weight is gained and soften with weight loss. (11) Recent evidence suggests this to hold true in overweight and obese middle-aged individuals (12, 13). It has been shown that when matched for age, PWV is ∼0.5 m/s higher in obese compared with non-obese. This increase in PWV is equivalent to 5-10 years of ageing (14). It has also been shown that aerobic physical activity is associated with the prevention of arterial stiffening (15), and moreover that aerobic training can reduce arterial stiffness (16).

The measurement of PWV is generally accepted as a simple, non-invasive, robust, and reproducible method to determine arterial stiffness and is considered to be the golden standard method for evaluation of arterial stiffness (17, 18). To the best of our knowledge, the current study is the first to investigate how a low-calorie diet (LCD) and intensive lifestyle intervention (ILI) affect arterial stiffness in a morbidly obese population.

The primary objective of this study was to compare the effect of a LCD and an ILI program on arterial stiffness in morbidly obese individuals. Our main hypothesis was that a 7-week ILI program consisting of aerobic endurance training 3 days a week and a moderate daily caloric restriction would have a greater effect on arterial stiffness than a 7-week LCD.

We further hypothesized that the loss of skeletal muscle mass and fat mass would be greater in the group subjected to a LCD.

## Methods

### Study design

This is the first part of a nonrandomized clinical trial comparing the effects of an intensive lifestyle modification program, a LCD, and bariatric surgery on arterial stiffness in morbidly obese patients. Results from the baseline cross-sectional analysis have been published elsewhere (19). (ClinicalTrials.gov Identifier NCT00626964).

### Setting

The study was performed at a tertiary care centre (the Morbid Obesity Centre, Vestfold Hospital Trust, Tønsberg, Norway) between February 2008 and February 2011. Before inclusion patients were either assigned to a comprehensive lifestyle modification program at the Clinic of Physical Medicine and Rehabilitation, Vestfold Hospital Trust, or to a LCD followed by bariatric surgery at the Morbid Obesity Centre.

### Participants

All participants were recruited from our tertiary care centre and had to reside within 100 km of either the hospital for rehabilitation or our clinic. The patients in the ILI group were all selected from subjects that had registered for participation in a standardized health promotion and weight reduction program at the hospital for rehabilitation. Participants in the LCD group were selected from patients due to undergo bariatric surgery at our hospital. The decision regarding the type of intervention was made prior to inclusion to our study and was not a part of our protocol.

Inclusion criteria were body mass index (BMI) ≥ 40.0 kg/m^2^ or BMI ≥ 35.0 kg/m^2^ accompanied by one or more obesity-related comorbidities.

Exclusion criteria were uncompensated heart failure, cardiac arrhythmias, unstable angina, end-stage renal disease, known bleeding disturbances, serious psychiatric disorders, serious eating disorders, cardiac pacemakers, intra-cardiac devices, cerebrovascular event, or a myocardial infarction within the last 6 months.

The study was approved by the regional ethics committee of the Southern Norway Regional Health Authority (code: S-05175) and was performed in accordance with the Declaration of Helsinki (20). Written informed consent was provided by all participants.

### Intensive lifestyle intervention

Each of the ILI groups comprised of 12-14 patients. The baseline individual energy expenditure was calculated according to Schofield's equation (21). Schofield's equation uses information pertaining to the patient's gender, age, and body weight to calculate resting metabolic rate (BMR). To calculate total energy expenditure, BMR was multiplied with a physical activity factor of 1.4, which is considered the increased energy cost of sedentary behavior (22). All participants received a dietary plan with an energy restriction of 1000 kcal/day in relation to calculated baseline energy expenditure, otherwise according to Norwegian nutritional guidelines (23).

Individuals in the ILI group were followed for 3 days per week during an intervention period of 7 weeks. Each day of intervention lasted 6 h and included two 90-min supervised training sessions, educational sessions regarding nutrition and physical activity. The first of the two supervised exercise sessions consisted of weight-bearing activities, such as walking, Nordic-walking, running, ballgames, resistance training, and other various exercises. The second exercise session consisted of water aerobics, swimming, and other water-based physical activities. The main aspect of the physical training was aerobic endurance training with moderate (four metabolic equivalents of task (METs)) to high intensity (eight METs). The resistance training was performed with 15-20 repetitions per exercise (24).

Study participants also had individual sessions with qualified personnel who used a client-centered counseling style known as motivational guidance techniques (25) to invoke behavioral change within the participants.

### Low calorie diet intervention

The LCD consisted of crisp bread combined with low-fat products for breakfast, lunch, and supper. Dinner consisted of fish, poultry, or lean meat combined with potatoes, rice, or pasta and vegetables. These ingredients did not provide >900 kcal per day, of which 37% of the energy came from protein, 43% from carbohydrates, and 20% from fat. Patients were allowed to increase the size of their meal using vegetables and water. Daily intake of a multivitamin/mineral supplement was recommended. Participants in the LCD group were instructed to maintain their habitual physical activity level throughout the intervention. The study participants were not allowed to attend other dietary or physical interventions prior to or during the 7 weeks of intervention in the present study.

### Outcomes

The main outcome variable was the 7-week change in arterial stiffness measured by PWV. Secondary outcomes were changes in weight, body composition, systolic and diastolic blood pressure, and pulse pressure.

### Data sources and measurements

All participants underwent a medical examination performed by a physician and a trained nurse. All measurements were performed after an overnight fast. Participants were instructed to withdraw from their prescribed medications and smoking prior to the examination on the day of testing. Blood was collected by venipuncture on the day of medical examination. Weight and height were measured with patients wearing light clothing and no shoes. BMI was calculated as weight in kilograms divided by the square of the height in meters. Waist circumference (WC) was measured midway between the 12th rib and the iliac crest. Blood pressure was measured after 5 min of rest using an electronic auscultatory blood pressure recorder with an appropriately sized cuff based on an arm circumference measurement (Dinamap®, ProCare Series, G.E. Medical Systems) with the patient sitting in an upright position. Three measurements were recorded. The average of the second and third recordings was used in the blood pressure analysis. Bioelectrical impedance measures were collected using the Inbody 720, Body Composition Analyzer, Biospace Co. Ltd. Arterial hypertension was defined by either, a systolic blood pressure ≥140 mmHg, diastolic blood pressure ≥90 mmHg, or the use of antihypertensive medication. Ischemic heart disease was defined as a history of stable coronary artery disease, percutaneous coronary intervention, coronary artery bypass graft surgery, or myocardial infarction. Mean arterial pressure (MAP) was calculated as [(diastolic pressure × 2) + systolic pressure]/3 (11).

The Sphygmocor system (Artcor, Sidney, Australia) and a single high-fidelity applanation tonometer (Millar®) were used to measure PWV. Pulse waves were obtained sequentially from the carotid and femoral artery. The locations of the sternal notch, carotid pulse, and femoral pulse were located. We placed tape directly above these spots with the patient in horizontal position to get a true linear measure. The distance was calculated as the distance between the carotid notch and the femoral artery minus the distance between the carotid notch and the carotid artery (18). The PWV was calculated from the transit time and the distance between these two arterial sites, determined in relation to the R-wave of the ECG, with patients lying in a horizontal position. Blood pressure was measured before every recording and entered into the Sphygmocor recorder to secure valid recordings with similar blood pressure. Three complete sets of data were sampled, and the average value was used as result.

Bioelectrical impedance analysis was obtained using Inbody 720, Body Composition Analyzer, Biospace Co. Ltd. Each patient was undressed except for underwear and placed in an upright position on the body composition analyzer. All jewelry and wrist-watches were removed before recording. The InBody 720 uses the segmental BIA method to examine the body as five cylinders (four limbs and a trunk) and measures impedance in these parts separately. It also uses electrical current at multi-frequency (5, 50, 250, 500, and 1000 kHz) to estimate the amount of extracellular and intracellular water. Homeostasis Model Assessment Insulin Resistance (HOMA- IR) was calculated as [fasting serum glucose (mmol/L) × fasting serum insulin (pmol/L)]/135 (26). Low-density lipoprotein cholesterol (LDL) concentrations were estimated by the Friedewald equation: LDL cholesterol = Total cholesterol − HDL cholesterol − (0.45 × triglycerides) (27). LDL cholesterol was not calculated if *S*-triglycerides were <0.2 mmol/L or ≥5 mmol/L.

### Laboratory analyses

Analyses of serum glucose and blood lipids were performed using dry reagent slide technology on the Vitros FS 5.1 (Ortho-Clinical Diagnostics, New York). Glycated hemoglobin (HbA1c) was analyzed using high-performance liquid chromatography on Tosoh HLC-723 G7 (Tosoh Corporation, Tokyo, Japan). Sera for analysis of insulin were stored at −20°C and analyzed within 1 week of blood sampling (Linco Research Inc., St. Charles, MO).

### Sample size

The present study is the first part of an ongoing non-randomized clinical trial. The sample size was calculated based on Barinas-Mitchell et al.'s (28) study, which showed that a mean weight reduction of 8% resulted in 1.4 m/s mean reduction in PWV in overweight individuals with diabetes. From this, we hypothesized that the mean PWV would be 8.2 m/s in both groups at baseline and that a 1-year weight loss of 8% in the ILI group would lead to a mean (SD) of 1.4 (2.8) m/s reduction in PWV. The expected weight loss following gastric bypass surgery is roughly three times as high, and we assumed that the mean reduction in PWV would be 2.8 m/s. Accordingly, at least 120 individuals had to complete the study to show a statistically significant difference between the two groups at a power of 80% (α = 0.05). To allow for a drop-out rate of <40%, we decided to include 200 patients (100 in each group).

### Statistical methods

Data are presented as mean (SD) or number (%) unless otherwise specified. Skewed data were transformed using natural logarithms to approximate normality. Differences between groups at baseline were analyzed using independent samples *t*-test or Fisher's exact test. Spearman's rank correlation was used to assess the bivariate association between weight loss, the change in fat mass, and the change in PWV. Within-group changes from baseline to follow-up were analyzed using paired samples *t*-test. Differences between groups in PWV at follow-up were analyzed using analysis of covariance (ANCOVA) including age, gender, MAP, baseline BMI, history of coronary artery disease, and baseline value of the dependent variable as covariates (11). Directed acyclic graphs (29) and baseline differences between groups were used as regression modeling tools to identify the confounding factors to be included as covariates in the ANCOVA. The model was further adjusted for change in MAP and reduction in weight to explore if the specific intervention was independently associated with the measured effect variable beyond the effect mediated by weight loss or reduction in MAP. Differences between groups in systolic blood pressure, diastolic blood pressure, and pulse pressure were analyzed using ANCOVA including age, gender, history of coronary artery disease, baseline BMI, and baseline value of dependent variable as covariates.

## Results

**FIGURE 1:**
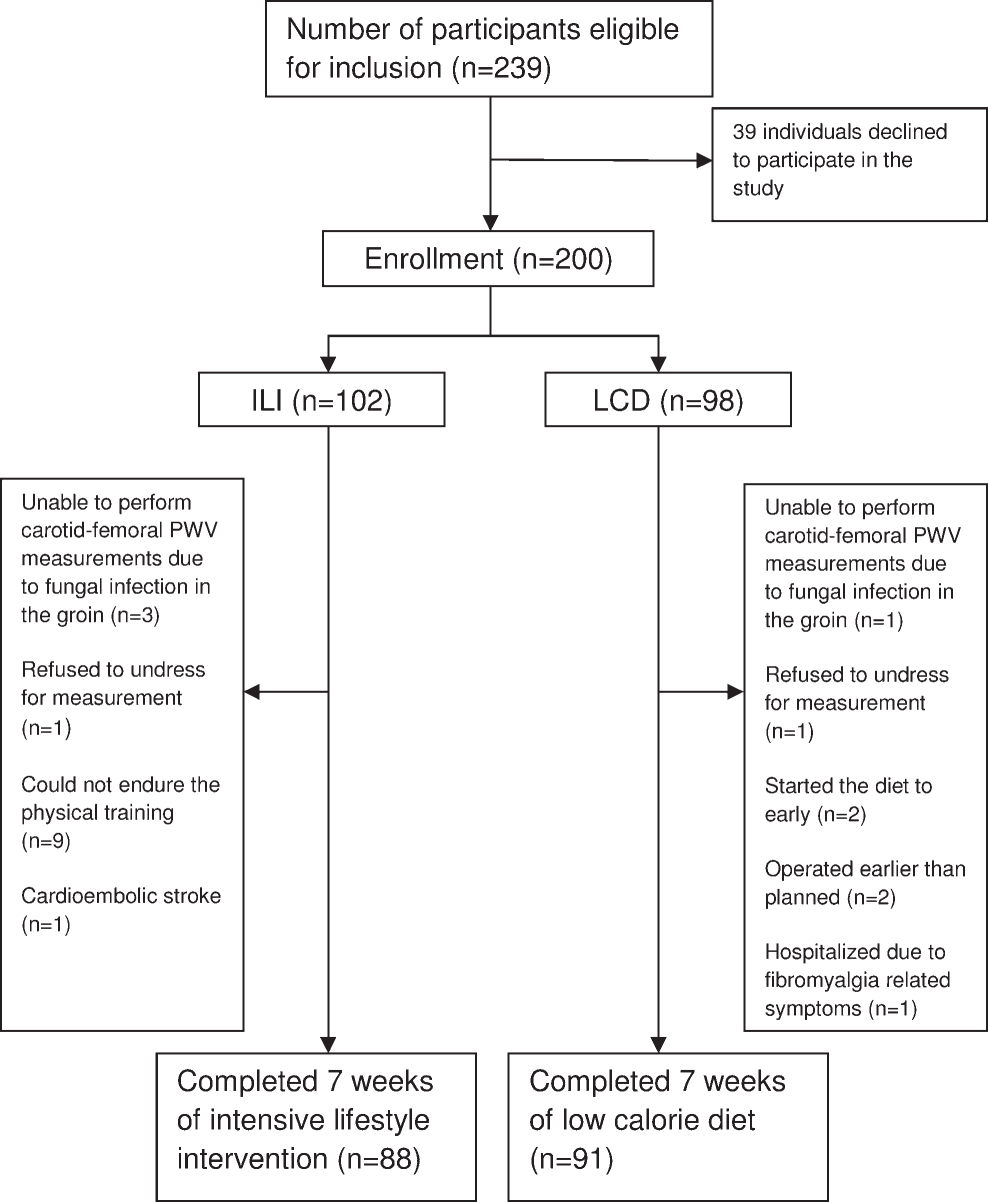
Flow chart.

During the inclusion period, 239 individuals were eligible for inclusion, 39 declined to participate leaving 200 individuals who accepted our invitation to participate in the study (Figure 1). Nine individuals in the ILI group found the activity level too demanding and withdrew from the study. Twelve individuals did not complete the study due to other reasons, leaving a total of 179 patients in the present analysis, 88 (56 women) in the ILI group and 91 (57 women) in the LCD group (Figure 1).

The groups were not significantly different in terms of PWV, but individuals in the LCD group were slightly younger and had higher systolic blood pressure, pulse pressure, slightly higher body weight and fat mass, and lower prevalence of ischemic heart disease compared with the ILI group (Table 1). Our records show that 40 (46%) of the individuals in the ILI group and 31 (34%) in the LCD group used antihypertensive medication at baseline, *P* = 0.129. The majority (39 out of 40 and 30 out of 31) were still taking antihypertensive medication at follow up.

**TABLE 1:** Baseline demography and differences between groups in anthropometric measures, carotid-femoral PWV, and blood pressure, medication, and glucose and lipid metabolism

	ILI	LCD	*P*
Age	45.2 (10.9)	42.3 (9.6)	0.045
Male/female	32/56	34/57	0.999
Diabetes	19 (21)	25 (27)	0.388
Hypertension	68 (77)	59 (65)	0.072
Ischemic heart disease	12 (14)	2 (2)	0.005
Smokers	14 (16)	18 (20)	0.560
*Weight and anthropometric measures*			
Weight, kg	125.2 (20.4)	137.8 (22.4)	≤0.001
Body mass index, kg/m^2^	42.5 (5.1)	45.7 (5.5)	≤0.001
Waist circumference, cm	128 (13)	133 (13)	0.007
Fat mass, kg	58.3 (12.4)	66.8 (12.9)	≤0.001
Skeletal muscle mass, kg	37.7 (7.3)	39.8 (8.0)	0.078
*PWV and blood pressure*			
Carotid-femoral pulse wave velocity, m/s	8.6 (1.8)	8.7 (1.7)	0.837
Systolic blood pressure, mm Hg	146 (17)	139 (19)	0.007
Diastolic blood pressure, mm Hg	80 (11)	81 (15)	0.613
Pulse pressure, mm Hg	66 (16)	58 (16)	0.002
*Medication*			
β-Blocker	14 (16)	11 (12)	0.521
Calcium-channel blocker	10 (11)	11 (12)	0.999
Inhibitors of the RAA system	34 (39)	26 (29)	0.159
Diuretics	18 (21)	12 (13)	0.232
Statins	19 (22)	12 (13)	0.168
*Glucose and lipid metabolism*			
Insulin, pmol/L	94.02 (53.2)	117.6 (100.1)	0.060
Glucose, mmol/L	6.0 (2.2)	6.1 (2.1)	0.516
HbA1C, %	6.0 (1.2)	6.1 (1.2)	0.789
HOMA-IR	4.09 (2.76)	5.65 (5.97)	0.057
Total cholesterol, mmol/L	5.1 (1.0)	4.9 (0.9)	0.156
LDL cholesterol, mmol/L	3.1 (1.0)	3.1 (0.8)	0.611
HDL cholesterol, mmol/L	1.2 (0.3)	1.1 (0.3)	0.074
Triglycerides, mmol/L	1.8 (1.2)	1.6 (0.9)	0.703

Number (%) or mean (SD). Independent samples *t*-test or Fischer's exact test.

### Changes in arterial stiffness and blood pressure

**FIGURE 2:**
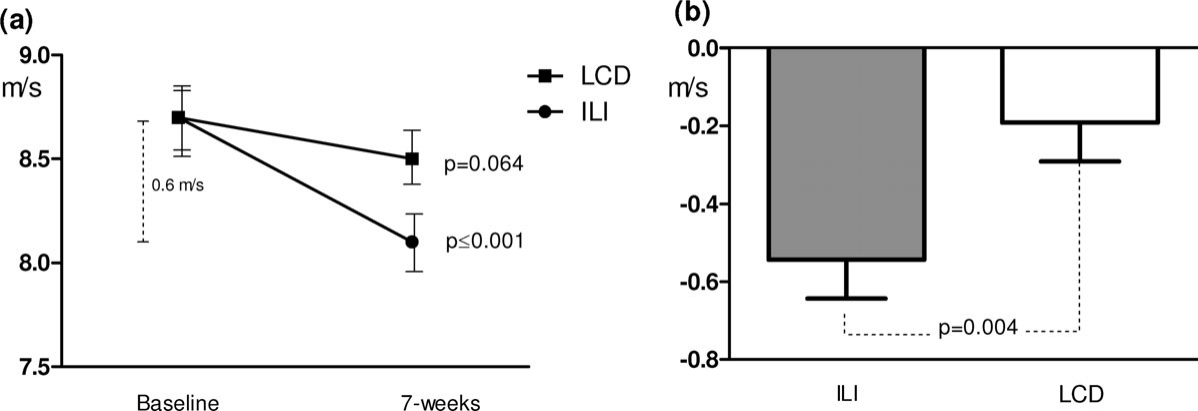
Changes in carotid-femoral PWV after 7 weeks of follow up. Within groups (**a**), adjusted difference between groups (**b**).

The ILI group had a statistically significant reduction in mean (95 % CI) PWV of −0.6 (−0.8, −0.4) m/s, *P* ≤ 0.001 (Figure 2a). In contrast, the reduction in PWV observed in the LCD group was not statistically significant, −0.2 (−0.4, 0.0) m/s, *P* = 0.064. After adjustment for age, gender, baseline MAP, baseline BMI, history of coronary artery disease, and baseline PWV, the decline in PWV was significantly larger in the ILI group than in the LCD group, between-group difference 0.4 (0.1, 0.6) m/s, *P* = 0.004 (Figure 2b). The larger decline in PWV observed in the ILI group (compared to the LCD group) was slightly attenuated but still significant after further adjustment for change in weight and change in MAP 0.3 (0.1, 0.6) m/s, *P* = 0.019.

**FIGURE 3:**
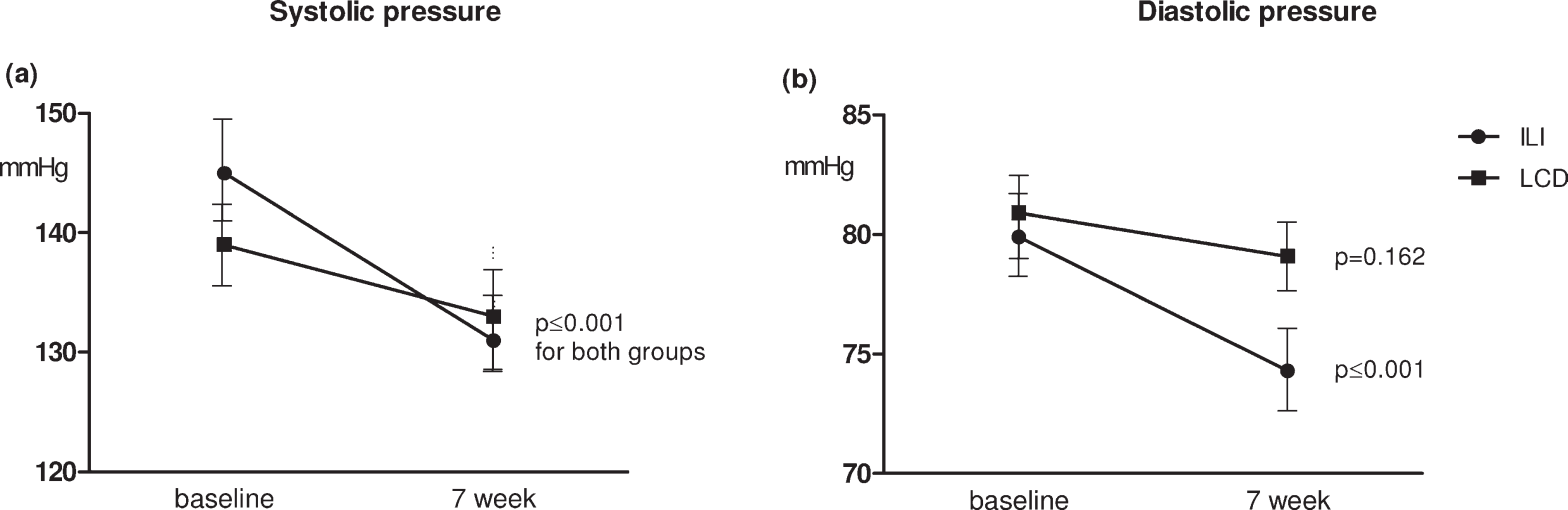
The within-group changes in systolic and diastolic blood pressure after 7 weeks of follow-up.

Both interventions led to a statistically significant reduction in systolic blood pressure, but only the ILI group showed a significant reduction in diastolic pressure (Figure 3a and b). When compared to the LCD group, the ILI group showed a larger decline in systolic and diastolic blood pressure after adjustments for age, gender, baseline BMI, history of coronary artery disease, baseline value of the dependent variable, and between-group difference: 5 (1, 9) and 5 (2, 7) mmHg; *P* = 0.038 and *P* ≤ 0.001, respectively. The same adjustments were made in terms of decline in pulse pressure and showed no significant difference between groups, *P* = 0.661 Table 2.

### Weight, body composition, glucose metabolism, and lipids

**FIGURE 4:**
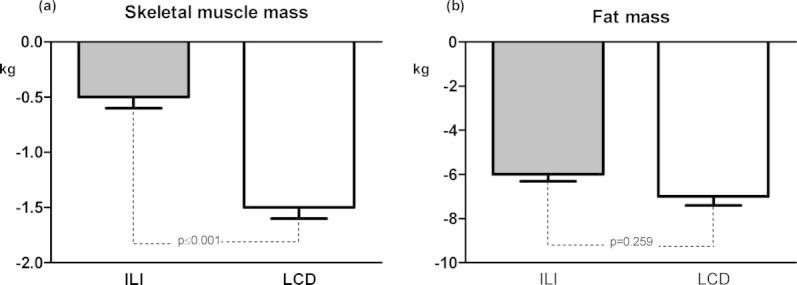
The loss of skeletal muscle mass and fat mass after 7 weeks of follow-up.

Body weight and WC were significantly reduced after both interventions (Table 3). After adjustments for age, gender, and baseline weight, a significantly larger weight loss was shown in the LCD group, between-group difference of −1.5 (−2.6, −0.5) kg, *P* = 0.006. In contrast, the adjusted reduction of WC was not significantly different between groups, *P* = 0.471. Bioelectrical impedance analysis showed a significant reduction in fat mass [−6.0 (−6.6, −5.3) in the ILI group and −7.0 (−7.8, −6.3) in the LCD group] and skeletal muscle mass [−0.5 (−0.7, −0.3) in the ILI group and −1.4 (−1.7, −1.2) in the LCD group], all *P* ≤ 0.001. After adjustments for age, gender, and baseline value of dependent variable, the loss of skeletal muscle mass was 0.8 (0.5, 1.1) kg larger in the LCD group, *P* ≤ 0.001, whereas no differences were apparent in terms of fat mass (Figure 4). Simple correlation analysis showed no significant associations between change in PWV and the changes in weight (*r* = −0.017, *P* = 0.821) and loss of fat mass (*r* = 0.041, *P* = 0.590). There were reduced blood glucose levels for both interventions with no statistically significant differences between groups (Table 3). There was a significantly greater decline in total-cholesterol and triglyceride levels and a smaller decline in HDL-cholesterol for patients in the ILI group compared to those in the LCD group#tbl^3^.

**TABLE 2:** Adjusted within- and between-group differences in carotid-femoral pulse wave velocity and blood pressure variables

	ILI	LCD	Adjusted difference	*P* value
PWV, m/s	−0.6 (−0.8, −0.4)	−0.2 (−0.4, 0.0)	−0.4 (−0.6, −0.1)	0.004
Systolic blood pressure, mm Hg	−13 (−16, −11)	−8 (−11, −5)	−5 (−9, −1)	0.038
Diastolic blood pressure, mm Hg	−6 (−8, −4)	−1 (−3, 1)	−5 (−7, −2)	≤0.001
Pulse pressure, mm Hg	−6 (−9, −3)	−7 (−10, −4)	−1 (−5, −3)	0.661

Mean (95% CI). Analyses of covariance (ANCOVA) including age, gender, baseline BMI, baseline MAP, history of coronary artery disease, and baseline status in dependent variable as covariates for PWV and age, gender, baseline BMI, history of coronary artery disease, and baseline status in dependent variable as covariates for systolic blood pressure, diastolic blood pressure and pulse pressure.

**TABLE 3:** The change in weight, anthropometric measurements, glucose, and lipid metabolism after 7 weeks of ILI or LCD

	ILI group	LCD group	Adjusted difference between groups	*P* value
Weight, kg	−6.6 (3.5)†	−9.4 (4.3)†	1.5 (0.5, 2.9)	0.006
BMI, kg/m^2^	−2.2 (1.4)†	−3.1 (1.3)†	0.6 (0.2, 0.9)	0.003
Waist circumference, cm	−6 (4)†	−7 (4)†	0.5 (−0.8, 1.8)	0.471
*Glucose metabolism*				
Insulin, pmol/L	−35.9 (37.1)†	−43.4 (82.1)†	9.3 (−0.6, 19.1)	0.065
Glucose, mmol/L	−0.5 (1.3)†	−0.5 (1.5)†	0.0 (−0.0, 0.0)	0.629
HbA1C, %	−0.4 (0.5)†	−0.4 (0.6)†	0.0 (−0.1, 0.1)	0.894
HOMA-IR	−1.8 (2.0)†	−2.5 (4.7)†	0.4 (−0.05, 0.9)	0.083
*Lipid metabolism*				
Total cholesterol, mmol/L	−0.6 (0.6)†	−0.4 (0.6)†	−0.2 (−0.4, 0.0)	0.032
LDL cholesterol, mmol/L	−0.3 (0.6)†	−0.2 (0.5)*	−0.1 (−0.3, 0.1)	0.193
HDL cholesterol, mmol/L	−0.1 (0.2)*	−0.1 (0.2)†	0.1 (0.0, 0.1)	0.007
Triglycerides, mmol/L	−0.5 (0.8)†	−0.3 (0.7)†	−0.2 (−0.3, −0.0)	0.009

Mean (SD or 95% CI).

* *P* ≤ 0.050,

† *P* ≤ 0.001. Paired samples *t*-test and ANCOVA adjusting for age, gender and baseline value of dependent variable.

## Discussion

Our findings demonstrate that in morbidly obese individuals, a comprehensive 7-week ILI program consisting of a moderate caloric restriction (1000 kcal/day) and aerobic physical activity of moderate to high intensity (4-8 METs) had a larger effect on arterial stiffness than a 7-week LCD (<900 kcal/day) intervention. The greater weight loss in the LCD group was due mainly to a significantly greater loss of skeletal muscle mass. No significant between group differences were evident in terms of the decline in fat mass or the reduction in WC.

### Comparison with the previous studies

Some prior studies have shown that PWV can be reduced in less obese through short-term (6 weeks to 3 month) weight reduction by the separate use of diets (12, 13) and increased aerobic physical exercise (16) or a combination of the two (30). To our knowledge, there are no studies exploring how arterial stiffness can be modified in a population of morbidly obese subjects.

The beneficial results of ILI on PWV are in accordance with the findings of a 1-year randomized clinical trial, which evaluated the effect of a behavioral weight loss intervention program combined with either orlistat or placebo in 38 obese individuals [average BMI 34.0 (5.2) kg/m^2^] with type 2 diabetes (28). The intervention included an energy restriction of ∼500 kcal per day and participants were encouraged to undertake 40-60 min of moderate intensity physical activity (such as walking or cycling) most week days. Independent of orlistat, the intervention was associated with a mean weight reduction of 7.8% (10 kg) and a 0.5 m/s reduction in PWV. As in the present study, the changes in PWV did not correlate significantly with weight loss. Importantly, this study differed from our own by the inclusion of relatively few morbidly obese subjects, no supervised training sessions, and a longer follow-up period.

Partly in contrast with our results, a short-term (12 weeks) randomized controlled study of overweight and obese healthy non-smoking middle-aged volunteers showed that a hypocaloric diet intervention (1200-1500 kcal/day) with no emphasis on physical activity was associated with a 7 kg reduction in weight and a significant reduction in PWV of 1.3 m/s (12). Although the weight loss was comparable, the effect on arterial stiffness was more pronounced than that in our study. It is hard to find a plausible explanation for this large difference, particularly in view of the higher level of physical activity in our ILI group. However, direct comparison between the studies is difficult given by differences in study design, time of follow up, and various degree of obesity among the participants.

### Arterial stiffness and insulin sensitivity and biological age

Arterial stiffness has been linked to age-related decline in muscle mass (31, 32). Loss of muscle mass is associated with reduced insulin sensitivity (33), and decreased insulin sensitivity is linked to arterial stiffness (34, 35). Individuals in the ILI group conserved more skeletal muscle mass than those in the LCD group. However, the greater loss of muscle mass in the LCD group compared to the ILI group did not translate into significant differences in terms of improvements in insulin sensitivity. The current popular notion that, in obese patients, a hypocaloric diet alone can reduce arterial stiffness could not be verified in Norwegian morbidly obese patients as the short-term LCD used failed to show any significant effect on arterial stiffness. Accordingly, the improved results in the ILI group are more likely to be mediated by the physical activity undertaken by individuals in that group.

Arterial stiffness is regarded as a measure of premature vascular ageing. According to the arterial stiffness reference value (36), the baseline biologic vascular age of our study population may be estimated to be 10-12 years older than it would be in normal-weight healthy individuals of a similar age. The reduction in PWV observed in the ILI group may translate into a reversal of age-related arterial stiffening of 5 to 7 years (37). Our findings support the notion that in morbidly obese individuals caloric restriction and physical activity might be preferable to weight loss by a LCD alone. Whether the short-term decline in PWV translates into a long-term clinically significant improvement in cardiovascular risk remains to be seen.

There are some limitations within the present study, which should be acknowledged. The major weaknesses are the lack of randomization and short follow-up time. According to Norwegian guidelines (38), tertiary care centers are obliged to provide morbidly obese patients with an appropriate treatment choice, either conservative or surgical. Both treatment choices are publicly funded. Accordingly, we found it unethical to assign patients to bariatric surgery if they both preferred and qualified for a conservative lifestyle intervention program, and vice versa. Other limitations include the lack of data illustrating the patients' adherence to the prescribed calorie restriction in both groups, and physical activity in the LCD group, and the absence of a weight maintenance period before the follow-up examination.

In summary, the findings from the present study indicate that a moderate caloric restriction combined with aerobic physical exercise can significantly reduce arterial stiffness in morbidly obese individuals after only 7 weeks of intervention. A LCD proved to be less effective in reducing arterial stiffness and showed no significant effect in spite of a clinically significant weight loss. It did, however, have a beneficial effect on both glucose and lipid metabolism, highlighting that important cardiovascular risk factors can be rapidly modified in morbidly obese individuals through a LCD.

## References

[1] Finucane MM, Stevens GA, Cowan MJ (2011). National, regional, and global trends in body-mass index since 1980: systematic analysis of health examination surveys and epidemiological studies with 960 country-years and 9.1 million participants. Lancet.

[2] Melanson KJ, McInnis KJ, Rippe JM, Blackburn G, Wilson PF (2001). Obesity and cardiovascular disease risk: research update. [Review] [50 refs]. Cardiol Rev.

[3] Flegal KM, Carroll MD, Kit BK, Ogden CL (2012). Prevalence of obesity and trends in the distribution of body mass index among US adults, 1999–2010. JAMA.

[4] Freedman DS, Khan LK, Serdula MK, Galuska DA, Dietz WH (2002). Trends and correlates of class 3 obesity in the United States from 1990 through 2000. JAMA.

[5] Hall KD, Sacks G, Chandramohan D (2011). Quantification of the effect of energy imbalance on bodyweight. Lancet.

[6] McAuley PA, Blair SN (2011). Obesity paradoxes. J Sports Sci.

[7] Fogelholm M (2010). Physical activity, fitness and fatness: relations to mortality, morbidity and disease risk factors. A systematic review. Obes Rev.

[8] Laurent S, Boutouyrie P, Asmar R (2001). Aortic stiffness is an independent predictor of all-cause and cardiovascular mortality in hypertensive patients. Hypertension.

[9] Vlachopoulos C, Aznaouridis K, Stefanadis C (2010). Prediction of cardiovascular events and all-cause mortality with arterial stiffness: a systematic review and meta-analysis. [Review] [49 refs]. J Am Coll Cardiol.

[10] Lim HE, Park CG, Shin SH, Ahn JC, Seo HS, Oh DJ (2004). Aortic pulse wave velocity as an independent marker of coronary artery disease. Blood Press.

[11] Wildman RP, Farhat GN, Patel AS (2005). Weight change is associated with change in arterial stiffness among healthy young adults. Hypertension.

[12] Dengo AL, Dennis EA, Orr JS (2010). Arterial destiffening with weight loss in overweight and obese middle-aged and older adults. Hypertension.

[13] Rider OJ, Francis JM, Ali MK (2009). Beneficial cardiovascular effects of bariatric surgical and dietary weight loss in obesity. J Am Coll Cardiol.

[14] Wildman RP, Mackey RH, Bostom A, Thompson T, Sutton-Tyrrell K (2003). Measures of obesity are associated with vascular stiffness in young and older adults. Hypertension.

[15] Vaitkevicius PV, Fleg JL, Engel JH (1993). Effects of age and aerobic capacity on arterial stiffness in healthy adults. Circulation.

[16] Tanaka H, Safar ME (2005). Influence of lifestyle modification on arterial stiffness and wave reflections. Am J Hypertens.

[17] Mansia G, De BG, Dominiczak A (2007). 2007 ESH-ESC Guidelines for the management of arterial hypertension: the task force for the management of arterial hypertension of the European Society of Hypertension (ESH) and of the European Society of Cardiology (ESC). Blood Press.

[18] Laurent S, Cockcroft J, Van BL (2006). Expert consensus document on arterial stiffness: methodological issues and clinical applications. Eur Heart J.

[19] Nordstrand N, Gjevestad E, Dinh KN (2011). The relationship between various measures of obesity and arterial stiffness in morbidly obese patients. BMC Cardiovasc Disord.

[20] World Medical Association declaration of Helsinki (1997). Recommendations guiding physicians in biomedical research involving human subjects. JAMA.

[21] Schofield WN (1985). Predicting basal metabolic rate, new standards and review of previous work. Hum Nutr Clin Nutr.

[22] Ainsworth BE, Haskell WL, Herrman SD (2011). Compendium of Physical Activities: a second update of codes and MET values. Med Sci Sports Exerc.

[23] Nasjonalt råd for ernæring (2011). Kostråd for å fremme folkehelsen og forebygge kroniske sykdommer i Norge. Helsedirektoratet.

[24] Ainsworth BE, Haskell WL, Leon AS (1993). Compendium of physical activities: classification of energy costs of human physical activities. Med Sci Sports Exerc.

[25] Rubak S, Sandbaek A, Lauritzen T, Christensen B (2005). Motivational interviewing: a systematic review and meta-analysis. Br J Gen Pract.

[26] Matthews DR, Hosker JP, Rudenski AS, Naylor BA, Treacher DF, Turner RC (1985). Homeostasis model assessment: insulin resistance and beta-cell function from fasting plasma glucose and insulin concentrations in man. Diabetologia.

[27] Friedewald WT, Levy RI, Fredrickson DS (1972). Estimation of the concentration of low-density lipoprotein cholesterol in plasma, without use of the preparative ultracentrifuge. Clin Chem.

[28] Barinas-Mitchell E, Kuller LH, Sutton-Tyrrell K (2006). Effect of weight loss and nutritional intervention on arterial stiffness in type 2 diabetes. Diabetes Care.

[29] McNamee R Confounding and confounders. Occup Environ Med.

[30] Blumenthal JA, Babyak MA, Hinderliter A (2010). Effects of the DASH diet alone and in combination with exercise and weight loss on blood pressure and cardiovascular biomarkers in men and women with high blood pressure: the ENCORE study. Arch Intern Med.

[31] Abbatecola AM, Chiodini P, Gallo C (2011). Pulse wave velocity is associated with muscle mass decline: Health ABC study. Age (Dordr).

[32] Ochi M, Kohara K, Tabara Y (2010). Arterial stiffness is associated with low thigh muscle mass in middle-aged to elderly men. Atherosclerosis.

[33] Volpi E, Nazemi R, Fujita S (2004). Muscle tissue changes with aging. Curr Opin Clin Nutr Metab Care.

[34] Stehouwer CD, Henry RM, Ferreira I (2008). Arterial stiffness in diabetes and the metabolic syndrome: a pathway to cardiovascular disease. Diabetologia.

[35] Ferreira I, Boreham CA, Twisk JW (2007). Clustering of metabolic syndrome risk factors and arterial stiffness in young adults: the Northern Ireland Young Hearts Project. J Hypertens.

[36] (2010). Determinants of pulse wave velocity in healthy people and in the presence of cardiovascular risk factors: ‘establishing normal and reference values’. Eur Heart J.

[37] Avolio AP, Chen SG, Wang RP, Zhang CL, Li MF, O'Rourke MF (1983). Effects of aging on changing arterial compliance and left ventricular load in a northern Chinese urban community. Circulation.

[38] Jakobsen GS, Hofso D, Roislien J, Sandbu R, Hjelmesaeth J (2010). Morbidly obese patients--who undergoes bariatric surgery?. Obes Surg.

